# Amniotic fluid embolism rescued using venoarterial extracorporeal membrane oxygenation without initial anticoagulation: A case report and literature review

**DOI:** 10.1097/MD.0000000000038176

**Published:** 2024-05-17

**Authors:** Hiroshi Araki, Motohiro Sekino, Yuri Hasegawa, Masaya Kurobe, Tetsufumi Motokawa, Akihiko Tanigawa, Takashi Egashira, Naoya Iwasaki, Miki Suzumura, Rintaro Yano, Sojiro Matsumoto, Taiga Ichinomiya, Ushio Higashijima, Naohiro Kanayama, Kiyonori Miura, Tetsuya Hara

**Affiliations:** a Department of Anesthesiology and Intensive Care Medicine, Nagasaki University Graduate School of Biomedical Sciences, Nagasaki, Japan; b Department of Obstetrics and Gynecology, Nagasaki University Graduate School of Biomedical Sciences, Nagasaki, Japan; c Department of Cardiovascular Medicine, Nagasaki University Graduate School of Biomedical Sciences, Nagasaki, Japan; d Department of Cardiovascular Surgery, Nagasaki University Hospital, Nagasaki, Japan; e Department of Obstetrics and Gynecology, Hamamatsu University School of Medicine, Hamamatsu, Japan.

**Keywords:** anticoagulants, blood coagulation disorders, DIC, disseminated intravascular coagulation, ECMO, pregnancy complication

## Abstract

**Rationale:**

Amniotic fluid embolism (AFE) is a fatal obstetric condition that often rapidly leads to severe respiratory and circulatory failure. It is complicated by obstetric disseminated intravascular coagulation (DIC) with bleeding tendency; therefore, the introduction of venoarterial extracorporeal membrane oxygenation (VA-ECMO) is challenging. We report the case of a patient with AFE requiring massive blood transfusion, rescued using VA-ECMO without initial anticoagulation.

**Patient’s concerns:**

A 39-year-old pregnant patient was admitted with a complaint of abdominal pain. An emergency cesarean section was performed because a sudden decrease in fetal heart rate was detected in addition to DIC with hyperfibrinolysis. Intra- and post-operatively, the patient had a bleeding tendency and required massive blood transfusions. After surgery, the patient developed lethal respiratory and circulatory failure, and VA-ECMO was introduced.

**Diagnosis:**

Based on the course of the illness and imaging findings, the patient was diagnosed with AFE.

**Interventions:**

By controlling the bleeding tendency with a massive transfusion and tranexamic acid administration, using an antithrombotic ECMO circuit, and delaying the initiation of anticoagulation and anti-DIC medication until the bleeding tendency settled, the patient was managed safely on ECMO without complications.

**Outcomes:**

By day 5, both respiration and circulation were stable, and the patient was weaned off VA-ECMO. Mechanical ventilation was discontinued on day 6. Finally, she was discharged home without sequelae.

**Lessons:**

VA-ECMO may be effective to save the lives of patients who have AFE with lethal circulatory and respiratory failure. For safe management without bleeding complications, it is important to start VA-ECMO without initial anticoagulants and to administer anticoagulants and anti-DIC drugs after the bleeding tendency has resolved.

## 1. Introduction

Amniotic fluid embolism (AFE) is a rare but life-threatening obstetric disorder, and the associated mortality rate is reported to be up to 43%.^[1,2]^ It occurs when the amniotic fluid, containing fetal cells and tissues, enters the maternal blood stream, resulting in severe respiratory and circulatory failure, and bleeding tendency due to disseminated intravascular coagulation (DIC) with hyperfibrinolysis.^[3]^

Venoarterial extracorporeal membrane oxygenation (VA-ECMO) is considered for use in fatal cases; however, hemorrhagic complications of the procedure are critical issues.^[4]^ Therefore, the induction of ECMO for AFE is not recommended in the existing guideline.^[3]^ In recent years, ECMO with an antithrombotic circuit has become available for clinical use, and it is possible to perform ECMO without anticoagulation in patients with AFE; however, no therapeutic strategy has been established in this regard.

In this report, we describe a case of AFE with lethal respiratory and circulatory failure, in which VA-ECMO was used to successfully manage the patient without serious complications, while controlling the bleeding tendency by a massive transfusion, using the antithrombotic ECMO circuit, and delaying the initiation of anticoagulant and anti-DIC drug administration.

## 2. Case report

A 39-year-old patient who was 25 weeks pregnant, gravida 5, para 2, was admitted to our hospital with complaint of abdominal pain. Her medical history included multiple uterine fibroids, persistent trophoblastic disease, and hypothyroidism. All coagulation tests on admission were not measurable; in addition, the platelet count was low (Table 1), and the Japanese Association for Acute Medicine DIC score^[5]^ was 7. The cause of DIC with hyperfibrinolysis was unknown at the time of admission, and during the examination process, a sudden decrease in fetal heart rate was observed. Therefore, she underwent emergency cesarean section under general anesthesia. The volume of intraoperative blood loss was 2104 mL (Table 1). Blood transfusions (red blood cell, 560 mL; frozen fresh plasma, 960 mL; cryoprecipitate, 300 mL; and platelet concentrate, 400 mL), 1 g of tranexamic acid (TXA), and surgical hemostasis were administered. However, the bleeding tendency continued, and the patient was transferred to the intensive care unit (ICU) under mechanical ventilation.

**Table 1:** The patient’s laboratory data, blood loss, and transfusion after intensive care unit admission.

Variables	ICU day										
	1				2	3	4	5	6	8	10
	Before ICU admission	At ICU admission	Pre-ECMO	Post-ECMO							
Hb (g/dL)	10.7	7.1	6.4	6.8	8.0	9.0	9.1	9.0	8.8	8.1	8.2
Plt (×10^4^/μL)	10.2	3.5	5.4	4.8	5.2	7.4	5.4	6.5	6.6	16.8	37.3
PT-INR	Unmeasurable	2.17	1.37	1.60	1.09	1.06	1.05	1.05	1.09	1.12	1.15
aPTT (s)	Unmeasurable	78.3	47.4	40.3	30.4	29.4	30.3	36.5	39.8	38.4	32.9
Fib (mg/dL)	Unmeasurable	37	101	91	188	200	166	150	156	263	372
FDP (μg/mL)	>960	>960	–	334.7	126.3	65.9	60.4	41.6	25.6	16.3	24.5
AT activity (%)	93	53	–	54	117	106	114	124	120	95	82
Lactate (mmol/L)	–	1.0	6.7	8.5	1.9	1.6	2.2	1.7	1.4	1.0	0.8
Blood loss (mL)	2104	577			99	97	3	205	12	0	0
RBC (mL)	560	1680			1120	280	280	0	0	0	0
FFP (mL)	960	4800			480	480	480	0	0	0	0
Cryoprecipitate (mL)	300	0			0	0	0	0	0	0	0
PC (mL)	400	800			400	0	0	0	0	0	0

aPTT = activated partial thromboplastin time, AT = antithrombin, ECMO = extracorporeal membrane oxygenation, FDP = fibrin/fibrinogen degradation products, FFP = fresh frozen plasma, Fib = fibrinogen, Hb = hemoglobin, ICU = intensive care unit, INR = international normalized ratio, PC = platelet concentrate, Plt = platelet, PT = prothrombin time, RBC = red blood cell concentrate.

**Figure 1.**
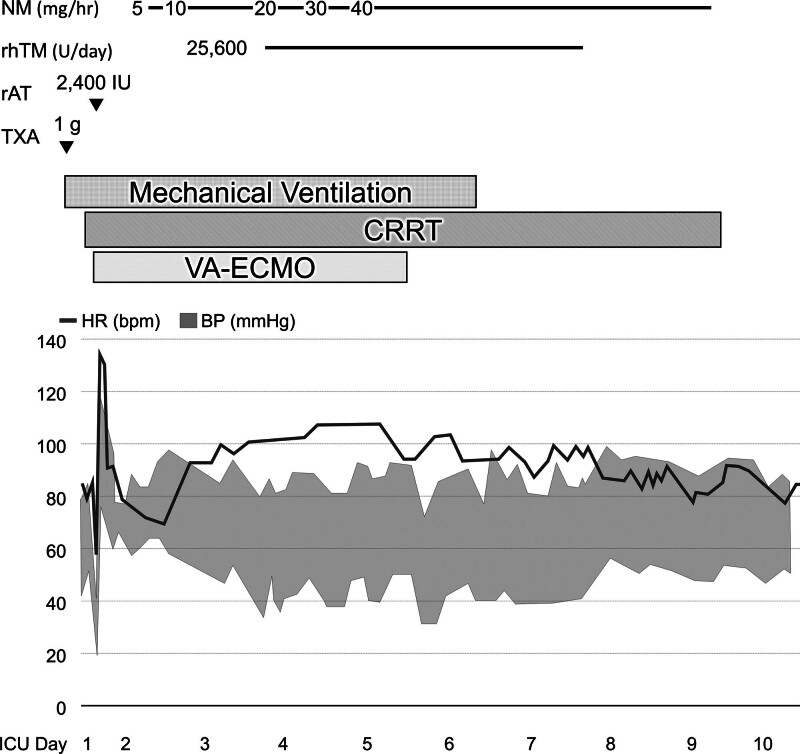
Clinical course, anticoagulation, and anti-DIC therapy after ICU admission. After admission to the ICU, an additional 1 g of TXA was administered along with blood transfusion. Three hours after admission, the patient’s blood pressure decreased and she developed oliguria, therefore CRRT was initiated. Four hours after admission, she developed lethal respiratory and circulatory failure under intensive care. VA-ECMO was introduced without anticoagulation. After bleeding tendency was controlled by massive transfusion, 2400 IU of rAT was administered for DIC treatment. In addition, NM for anticoagulation was started at a low dose of 5 mg/h on ICU day 2 and gradually increased; rhTM for DIC treatment at a dose of 25,600 IU was started on ICU day 3. By ICU day 5, both respiration and circulation were stable, and the patient was weaned off VA-ECMO. Mechanical ventilation was discontinued on ICU day 6. She was weaned off CRRT on ICU day 9 and transferred to a ward on ICU day 10. BP = blood pressure, CRRT = continuous renal replacement therapy, DIC = disseminated intravascular coagulopathy, HR = heart rate, ICU = intensive care unit, NM = nafamostat mesylate, rAT = recombinant antithrombin, rhTM = recombinant human soluble thrombomodulin, TXA = tranexamic acid, VA-ECMO = venoarterial extracorporeal membrane oxygenation.

**Figure 2.**
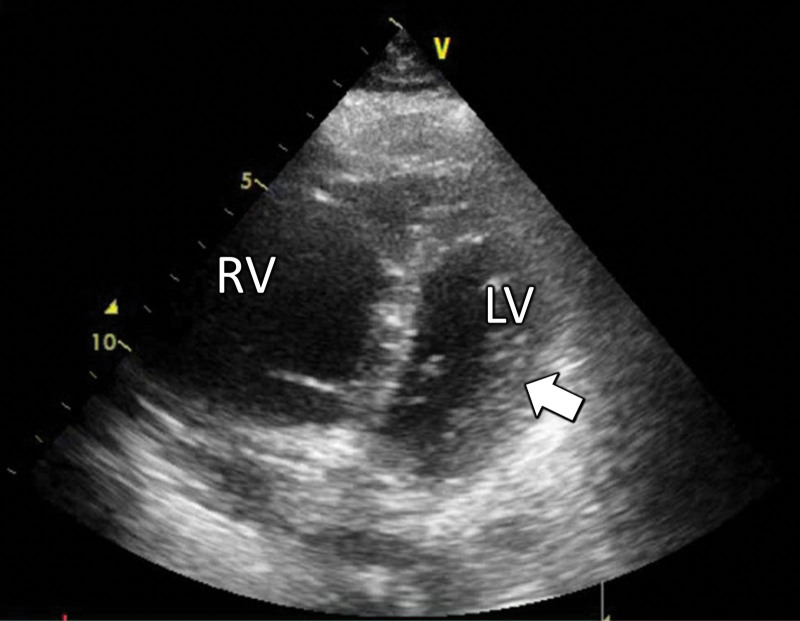
Parasternal short axis view on transthoracic echocardiography showing the marked enlargement of the right ventricle and collapse of the left ventricle (white arrow). LV = left ventricle, RV = right ventricle.

Upon admission to the ICU (ICU day 1), blood and coagulation tests showed severe anemia, persistent thrombocytopenia, and coagulopathy (Table 1). Additional 1 g of TXA was administered (Fig. 1), and blood transfusions were continued. Three hours after admission, the patient’s blood pressure decreased, and noradrenaline was commenced. As she developed oliguria, continuous renal replacement therapy (CRRT) was initiated without anticoagulation. Four hours after admission, the patient developed severe respiratory and circulatory failure. She required high doses of vasopressor and inotropic agents (noradrenaline, 0.5 μg/kg/min; vasopressin, 1.8 units/h; and adrenaline, 0.25 μg/kg/min), and her PaO_2_/FIO_2_ ratio and lactate level were 106 mm Hg and 6.7 mmol/L respectively. The parasternal short axis view on transthoracic echocardiography showed marked enlargement of the right ventricle and collapse of the left ventricle (Fig. 2 and Video S1, Supplemental Digital Content, http://links.lww.com/MD/M500), and AFE or pulmonary thromboembolism was suspected. Subsequently, we could not maintain her blood pressure despite the use of vasopressors and inotropes, including an intravenous bolus administration of adrenaline. Due to the possibility of bleeding exacerbation, VA-ECMO was introduced without anticoagulation. A venous drainage cannula (21 Fr) and an arterial return cannula (16.5 Fr) (both CAPIOX, percutaneous catheter; Terumo, Tokyo, Japan) were placed through the right femoral vein and left femoral artery for VA-ECMO, which was maintained with blood flow at 4.0 L/min using a centrifugal pump (CAPIOX Emergency Bypass System; Terumo) and membrane oxygenator (CAPIOX LX; Terumo). The blood-contacting surfaces of these devices were coated with poly-2-methoxyethyl acrylate (PMEA) (X Coating; Terumo) to reduce platelet adhesion to foreign surfaces and inhibit thrombus formation, thereby maintaining platelet count and function, and reducing blood loss and need for transfusion.^[6]^

Contrast-enhanced computed tomography showed no pulmonary thromboembolism, and a clinical diagnosis of AFE was made. We continued transfusions to maintain fibrinogen > 150 mg/dL and platelets > 50,000/μL (Table 1). After bleeding tendency was controlled, we administered 2400 IU of recombinant antithrombin (Kyowa Kirin Co., Ltd., Tokyo, Japan). Additionally, nafamostat mesylate (NM) (Sawai Pharmaceutical Co., Ltd., Osaka, Japan) was commenced at a low dose of 5 mg/h on ICU day 2 (18 hours after ECMO initiation) and gradually increased (Fig. 1). As fibrin degradation products levels were still high and signs of DIC persisted, recombinant human soluble thrombomodulin (rhTM) (Asahi Kasei Pharma Co., Ltd, Tokyo, Japan) at a dose of 25,600 IU was started on ICU day 3. There was no increase in bleeding after that. By ICU day 5, both respiration and circulation were stable, and the patient was weaned off VA-ECMO and mechanical ventilation on ICU day 6. She was weaned off CRRT on ICU day 9 and transferred to a ward on ICU day 10. Eleven days later, she was discharged home without sequelae. Her baby was treated in the neonatal ICU and growing care unit, and discharged home 3 months after birth.

Our case met the Japanese criteria of AFE and was diagnosed as clinical AFE.^[7]^ Moreover, auxiliary diagnosis of AFE was supported by serological methods. Intraoperative maternal serum analysis results, which were later determined, were as follows: zinc coproporphyrin-1 (Zn-CP1), 5.8 pmol/mL (normal, <1.6 pmol/mL); sialyl Tn antigen, 16.0 IU/mL (normal, <45 IU/mL); complement factor 3 (C3), 92 mg/dL (normal, 80–140 mg/dL); and C4, 21 mg/dL (normal, 11–34 mg/dL). Results at the commencement of ECMO were Zn-CP1, <1.6 pmol/mL; C3, 63 mg/dL; and C4, 9 mg/dL. Increased Zn-CP1 and decreased C3 and C4 strongly suggested a diagnosis of AFE.^[7–9]^

## 3. Discussion

Herein, we have reported a case of AFE that was salvaged using VA-ECMO. Coagulopathy is a critical problem in AFE, and the introduction of VA-ECMO may further exacerbate bleeding tendency. In this case, the initiation of anticoagulation was delayed and a massive transfusion was performed to prioritize hemostasis. VA-ECMO was safely maintained, which saved the patient; without ECMO, the patient could have died.

It has been reported from ECMO volume centers that VA-ECMO without unfractionated heparin saved the lives of 7 out of 10 patients who had fatal AFE with coagulopathy and bleeding.^[1]^ A recent retrospective study, which did not include patients with AFE, reported that VA-ECMO can be performed without anticoagulants for a median of 70 hours and that it reduces bleeding complications by using a heparin-coated circuit.^[4]^ Antithrombotic coating of blood contact surfaces of the ECMO circuit is one of the techniques used to maintain ECMO without systemic anticoagulation. The purpose of the coating is to confer antithrombotic properties, and there are 2 types of coating: bioactive and biopassive surface coatings.^[10]^ The heparin-coated circuit is the bioactive type. There is evidence that heparin-coated ECMO circuits reduce or eliminate systemic heparin dosing without adverse coagulation or inflammatory responses.^[11]^ However, heparin coatings, especially ion-bound heparin coatings, can leach into the bloodstream during long-term use.^[1]^ On the other hand, PMEA-coated circuits have biopassive coatings, and they reduce platelet adhesion, platelet aggregation, and protein adsorption.^[12]^ In a report comparing heparin-coated and PMEA-coated circuits in aortic arch surgery involving prolonged cardiopulmonary bypass, the PMEA-coated circuit required less platelet transfusion.^[13]^ However, there is no report comparing heparin-coated and PMEA-coated circuits in VA-ECMO.^[10]^ ECMO circuits with antithrombotic coatings may be particularly useful for patients concerned about bleeding and who want to avoid anticoagulation, such as in the present case of AFE.

For patients with AFE under ECMO, adjuvant therapies (such as blood transfusions, anticoagulation for ECMO, and DIC treatment after controlling bleeding tendency) are also important for improving the severe condition. DIC is classified into thrombotic and fibrinolytic phenotypes; the latter causes bleeding symptoms, and it is the type seen in AFE.^[14]^ TXA is an antifibrinolytic agent that prevents fibrin degradation by inhibiting the conversion of plasminogen to plasmin.^[15]^ When obstetric hemorrhage occurs, early TXA administration reduces the risk of associated mortality.^[16]^ A study reported that patients with AFE who died received significantly less TXA than those who survived did.^[17]^ On the other hand, for severe coagulopathy caused by AFE, it is important to initially control bleeding by replenishing platelets and coagulation factors through aggressive transfusion.^[3]^ It is recommended that platelet counts should be over 50,000/µL,^[3,18]^ and activated partial thromboplastin time and international normalized ratio should be normal or close to the normal range.^[3]^ Additionally, target fibrinogen level of at least 150 to 200 mg/dL is recommended for obstetric hemorrhage.^[19]^ In our case, bleeding tendency was controlled with intra- and post-operative TXA administration and aggressive massive blood transfusion.

Furthermore, in cases like the current case, after bleeding symptoms subside, and platelet counts and coagulation tests reach target values, administration of anticoagulants in order to maintain ECMO for a prolonged time and to prevent thrombotic complications, and anti-DIC drugs should be considered. While unfractionated heparin is the common anticoagulant used in ECMO,^[20]^ we used NM in our case. NM is a synthetic serine protease inhibitor, often used as an anticoagulant for patients with a high bleeding risk on CRRT.^[21,22]^ The advantage of NM is its short half-life of approximately 8 to 10 minutes, making it theoretically effective for patients with a bleeding tendency who are undergoing ECMO.^[23]^ For patients undergoing ECMO, there is one report that NM increases the risk of bleeding, compared with heparin;^[24]^ however, one recent report states the opposite.^[25]^ A case of successful use of NM as an anticoagulant in venovenous ECMO for management of AFE has been reported.^[26]^ In conditions such as AFE, where bleeding risk is high, NM may be a better anticoagulant choice because of its short half-life, which makes it easier to regulate anticoagulation; however, it should be started at low doses, as in this case.

Finally, intervention for DIC concomitant with AFE may also play an important role. There are reports that rhTM and AT are effective in the management of obstetric DIC with hyperfibrinolysis.^[27–29]^ By inhibiting excessive activation of thrombin, rhTM regulates an unbalanced coagulation system. In addition, rhTM has antifibrinolytic and anti-inflammatory properties that may alleviate some of the fatal pathophysiology of DIC.^[30]^ A complication of rhTM administration is bleeding. A recent meta-analysis reported a trend toward increased bleeding complications when rhTM was administered to patients with other than septic DIC,^[31]^ although the difference was not statistically significant. With respect to obstetric DIC, a study reported fewer platelet transfusion requirements, improved coagulation tests, and increased platelet counts in an rhTM-treated group compared with a control group.^[28]^ AT mainly acts by inhibiting thrombin; however, it inhibits several other coagulation factors and is expected to have anti-inflammatory effects.^[32]^ Although AT has not yet been proven to improve prognosis in patients with obstetric DIC, it may reduce the need for hysterectomy,^[29]^ which is an important issue for women of childbearing potential. In our case, after confirming that the bleeding tendency was under control, recombinant antithrombin and rTM were administered. Although these DIC drugs could be administered without any problems in the current case, caution should be exercised because they may exacerbate the bleeding tendency.

Despite the high mortality rate of AFE, no specific treatment for the condition has been established; VA-ECMO may be an effective life-saving intervention in fatal AFE; however, the actual recommended implementation of ECMO, as well as the details of transfusion, anticoagulation, and anti-DIC therapy, is unknown. Therefore, we collected case reports of patients with AFE who were managed with VA-ECMO, and investigated their characteristics, the implementation of ECMO, and treatment outcomes. The case reports were extracted through a search of PubMed using the following search formula: “Embolism, Amniotic Fluid” (medical subject headings [MeSH]) AND “Extracorporeal Membrane Oxygenation” [MeSH]. As a result, 19 patients were identified from 19 case reports,^[33–51]^ including the case report cited in the extracted article.^[43,46]^ Cases in which VA-ECMO was used only in the operating room or venovenous ECMO was used, were excluded. Finally, data from 20 patients, including the present case, were examined and reviewed.

Table 2 summarizes the details of initial anticoagulation (within 12 hours of ECMO initiation) and outcomes of patients with AFE treated using VA-ECMO. The patients’ median age was 35 (33–39) years. Fifteen patients had cardiopulmonary arrest before ECMO initiation. At least 9 patients were anticoagulated within 12 hours of ECMO initiation (bolus administration and/or continuous administration), and heparin was the anticoagulant used in the majority of patients. Five patients were not anticoagulated within 12 hours of ECMO initiation,^[39,40,47,48]^ whereas 3 patients who required ECMO for more than 4 days, including the patient in the present case, subsequently received anticoagulation.^[40,48]^ The use of antithrombotic ECMO circuits was described in only 4 cases, 3 of which were heparin-coated circuits^.[39,40,42]^ Anti-DIC treatment was not mentioned in any of the cases, except for the present case. Many case reports did not include details related to anticoagulation during ECMO, suggesting a possible lack of interest. On the other hand, 14 case reports (74%) described the need for massive transfusions (defined as transfusion of 10 ≥ units of red blood cells^[52]^ or when it was stated that massive transfusion was performed), indicating that bleeding tendency due to AFE and anticoagulation associated with ECMO are critical issues. In fact, at least in 3 cases,^[42,45,48]^ bleeding was reported to have occurred after anticoagulation initiation. The patients were weaned off ECMO in 3 days (2–4 days), and in-hospital survival was as high as 100%; however, publication bias is a serious concern. An observational study from 2 ECMO centers showed survival in 7 of 10 fatal cases with AFE that required ECMO.^[1]^ Additionally, a report from the Extracorporeal Life Support Organization Registry^[53]^ showed survival in 21 of 34 patients (relatively high survival rate of 62%). In other words, VA-ECMO may be a promising treatment option in patients with fatal AFE. However, the details of ECMO management vary from case to case. Further research on anticoagulation in the management of ECMO in patients with AFE is expected.

**Table 2:** Details of the initial anticoagulation and outcomes of patients with amniotic fluid embolism treated using venoarterial extracorporeal membrane oxygenation.

No	Age (years)	CPA before ECMO	Anticoagulation within 12 h		Anticoagulant	Coating of ECMO circuit	Massive transfusion	Duration of ECMO (d)	Maternal outcome	Author, year
			Bolus anticoagulation	Continuous anticoagulation						
1	34	Yes	–	Yes	Heparin	–	Yes	2	Survived	Hsieh et al, 2000^[33]^
2	21	Yes	–	–	–	–	–	2	Survived	Shen et al, 2009^[34]^
3	43	Yes	Yes	Yes	Heparin	–	Yes	1	Survived	Ecker et al, 2012^[35]^
4	35	Yes	–	–	–	–	–	3	Survived	Fang et al, 2016^[36]^
5	39	No	–	–	–	–	–	3	Survived	Wise et al, 2016^[37]^
6	39	Yes	No	Yes	Heparin	–	Yes	2	Survived	Huang et al, 2017^[38]^
7	32	Yes	No	No	None	Heparin	Yes	1	Survived	Seong et al, 2018^[39]^
8	35	No	No	No	Heparin	Heparin	No	4	Survived	Tincrès et al, 2018^[40]^
9	30	Yes	Yes	–	Heparin	–	Yes	2	Survived	Viau-Lapointe 2019^[41]^
10	34	Yes	No	No	Calcium citrate, heparin	Heparin	Yes	7	Survived	Eiras et al, 2019^[42]^
11	42	Yes	–	–	–	–	Yes	2	Survived	Gitman et al, 2019^[43,46]^
12	36	Yes	No	Yes	–	–	–	5	Survived	Depondt et al, 2019^[44]^
13	39	No	–	Yes	Heparin	–	Yes	5	Survived	Kim et al, 2020^[45]^
14	18	Yes	–	Yes	–	–	Yes	3	Survived	Chiao et al, 2020^[46]^
15	38	Yes	No	No	None	–	Yes	1	Survived	Ge et al, 2021^[47]^
16	40	Yes	No	Yes	Heparin	–	Yes	4	Survived	Adachi et al, 2021^[48]^
17	35	Yes	–	–	–	–	Yes	4	Survived	Wu et al, 2022^[49]^
18	31	No	–	–	–	–	Yes	5	Survived	Golzarian et al, 2023^[50]^
19	33	Yes	Yes	–	Heparin	–	Yes	1	Survived	Sundin et al, 2024^[51]^
20	39	No	No	No	Nafamostat	PMEA	Yes	5	Survived	2024 (our case)

AFE = amniotic fluid embolism, CPA = cardiopulmonary arrest, ECMO = extracorporeal membrane oxygenation, PMEA = poly-2-methoxyethyl acrylate.

In the current case, the blood transfusion volume and anticoagulant dose were adjusted using the commonly used coagulation tests; however, in recent years, case reports have emerged showing that point-of-care monitoring with thromboelastography and rotational thromboelastometry was useful in the management of patients with AFE (although ECMO was not used).^[54–56]^ Use of thromboelastography/rotational thromboelastometry may lead to more successful anticoagulation strategies and treatment of DIC secondary to AFE during VA-ECMO. Finally, ECMO can cause infections and mechanical complications in addition to bleeding.^[57]^ Thus, physicians should additionally be aware that the introduction of ECMO in inexperienced facilities may worsen patients’ outcomes.

In conclusion, VA-ECMO is effective for managing patients who have AFE with lethal circulatory and respiratory failure. For safe management without complications, it is important to start VA-ECMO without initial anticoagulants, administer massive transfusion and TXA, and use antithrombotic ECMO circuit in the early phase; then, anticoagulants and anti-DIC drugs should be administered after bleeding tendency resolves. However, further data accumulation and verification are needed to prove the effectiveness of this strategy.

## Acknowledgments

We would like to thank Editage (www.editage.jp) for English language editing.

## Author contributions

**Conceptualization:** Hiroshi Araki, Motohiro Sekino, Yuri Hasegawa, Masaya Kurobe, Tetsufumi Motokawa, Akihiko Tanigawa, Takashi Egashira, Naoya Iwasaki, Miki Suzumura, Rintaro Yano, Sojiro Matsumoto, Taiga Ichinomiya, Ushio Higashijima, Naohiro Kanayama, Kiyonori Miura, Tetsuya Hara.

**Data curation:** Hiroshi Araki, Motohiro Sekino.

**Formal analysis:** Hiroshi Araki, Motohiro Sekino.

**Investigation:** Hiroshi Araki, Motohiro Sekino, Yuri Hasegawa, Masaya Kurobe, Tetsufumi Motokawa, Akihiko Tanigawa, Takashi Egashira, Naoya Iwasaki, Miki Suzumura, Rintaro Yano, Sojiro Matsumoto, Taiga Ichinomiya, Ushio Higashijima, Naohiro Kanayama, Kiyonori Miura, Tetsuya Hara.

**Supervision:** Motohiro Sekino, Tetsuya Hara.

**Visualization:** Hiroshi Araki, Masaya Kurobe, Tetsufumi Motokawa.

**Writing – original draft:** Hiroshi Araki.

**Writing – review & editing:** Hiroshi Araki, Motohiro Sekino, Yuri Hasegawa, Masaya Kurobe, Tetsufumi Motokawa, Akihiko Tanigawa, Takashi Egashira, Naoya Iwasaki, Miki Suzumura, Rintaro Yano, Sojiro Matsumoto, Taiga Ichinomiya, Ushio Higashijima, Naohiro Kanayama, Kiyonori Miura, Tetsuya Hara.

## Supplementary Material
